# Human lung cDC1 drive increased perforin-mediated NK cytotoxicity in chronic obstructive pulmonary disease

**DOI:** 10.1152/ajplung.00322.2020

**Published:** 2021-10-27

**Authors:** Alexander M. Pallazola, Jessica X. Rao, Dawit T. Mengistu, Maria S. Morcos, Mariam S. Toma, Valerie R. Stolberg, Alexandra Tretyakova, Lisa McCloskey, Jeffrey L. Curtis, Christine M. Freeman

**Affiliations:** ^1^Pulmonary & Critical Care Medicine Division, Department of Internal Medicine, University of Michigan Medical School and Michigan Medicine, Ann Arbor, Michigan; ^2^Research Service, VA Ann Arbor Healthcare System, Ann Arbor, Michigan; ^3^Graduate Program in Immunology, University of Michigan, Ann Arbor, Michigan; ^4^Pulmonary and Critical Care Medicine Section, VA Ann Arbor Healthcare System, Ann Arbor, Michigan

**Keywords:** COPD, dendritic cells, human, natural killer cells, perforin

## Abstract

In chronic obstructive pulmonary disease (COPD), lung natural killer cells (NKs) lyse autologous lung epithelial cells in vitro, but underlying mechanisms and their relationship to epithelial cell apoptosis in vivo are undefined. Although this cytolytic capacity of lung NKs depends on priming by dendritic cells (DCs), whether priming correlates with DC maturation or is limited to a specific DC subset is also unknown. We recruited ever-smokers (≥10 pack-years; *n* = 96) undergoing clinically indicated lung resections. We analyzed lung NKs for cytotoxic molecule transcripts and for cytotoxicity, which we correlated with in situ detection of activated Caspase-3/7+ airway epithelial cells. To investigate DC priming, we measured lung DC expression of CCR2, CCR7, and CX3CR1 and cocultured peripheral blood NKs with autologous lung DCs, either matured using lipopolysaccharide (LPS) (nonobstructed smokers) or separated into conventional dendritic cell type-1 (cDC1) versus cDC type-2 (cDC2) (COPD). Lung NKs in COPD expressed more perforin (*P* < 0.02) and granzyme B (*P* < 0.03) transcripts; inhibiting perforin blocked in vitro killing by lung NKs. Cytotoxicity in vitro correlated significantly (*S_r_* = 0.68, *P* = 0.0043) with numbers of apoptotic epithelial cells per airway. In nonobstructed smokers, LPS-induced maturation enhanced DC-mediated priming of blood NKs, reflected by greater epithelial cell death. Although CCR7 expression was greater in COPD in both cDC1 (*P* < 0.03) and cDC2 (*P* = 0.009), only lung cDC1 primed NK killing. Thus, rather than being intrinsic to those with COPD, NK priming is a capacity of human lung DCs that is inducible by recognition of bacterial (and possibly other) danger signals and restricted to the cDC1 subset.

## INTRODUCTION

Chronic obstructive pulmonary disease (COPD) is a highly prevalent condition resulting from inhaled oxidants including cigarette smoke exposure (1). COPD is associated with airway remodeling, mucus hypersecretion, inflammatory cell infiltration, and tissue destruction, leading to largely irreversible lung function decline. Once lung damage begins, neither smoking cessation nor current therapies halt progression, making novel therapeutic approaches urgently needed. Apoptosis of lung structural cells is implicated in emphysema development (2–4), but responsible mechanisms are undefined.

Natural killer cells (NKs) are innate lymphocytes that rapidly induce apoptosis of abnormal cells, including those that are damaged, infected, or malignant. NKs from the sputum and alveolar fluid of subjects with COPD were more cytotoxic toward a target cell line than NKs from ever-smokers with no airway obstruction (NAO; 5, 6). We showed that lung NKs from subjects with COPD induced greater apoptosis of autologous lung epithelial cells in vitro than lung NKs from subjects with NAO, and that this difference depended on the effector lung NKs rather than their targets (7, 8). Human lung NKs primarily display the differentiated CD56^dim^CD16^+^ phenotype associated with potent cytotoxicity, but the frequency of this phenotype does not differ between ever-smokers without versus with COPD (8), arguing for additional differences in those with COPD. In addition, the mechanism by which lung NKs kill targets in COPD is unproven and important to define.

During homeostasis, human lung NKs require priming signals to transition to a cytotoxic state (9). Dendritic cells (DCs), which orchestrate immune responses (10), are crucial for this priming (7). In the lymph nodes of transgenic mice, IL-15 transpresentation by DCs was necessary and sufficient to prime resting NKs (11). We showed that human lung DCs increase the cytotoxic response of blood NKs toward autologous lung epithelial cells, against which they normally show weak cytotoxicity. This increased cytotoxicity was also mediated by IL-15Rα transpresentation and was greater in subjects with COPD than in smokers without airflow obstruction (7). Mature DCs are more capable than immature DCs of priming NKs (12, 13), and we previously demonstrated a correlation between increased lung DC maturation and spirometrically defined COPD severity (14). However, whether maturation of human lung DCs is mechanistically linked to their priming ability is unknown.

DCs can be classified into subsets, though understanding of their ontogeny and function continues to evolve rapidly. For this study, we focused on conventional dendritic cell type-1 and type-2 (cDC1 and cDC2, respectively). cDC1 drive Type 1 helper (Th1) responses and present apoptotic cell-associated antigens (15), whereas cDC2 are important to recruit other inflammatory cell populations (16). Whether a specific DC subset is required to prime lung NKs is unknown and crucial to answer to allow for tailored therapies. Hence, our goals were to define mechanisms of NK killing in COPD, to test whether NK cytotoxicity as assayed in vitro correlates with airway epithelial cell damage, and to determine the relationship of lung DC maturation state and subset to NK priming ability.

## MATERIALS AND METHODS 

### Ethics Statement

Studies and consent procedures were performed in accordance with the Declaration of Helsinki at the VA Ann Arbor Healthcare System and Michigan Medicine and were approved by both Institutional Review Boards (FWA00000348 and FWA00004969, respectively). Written informed consent was obtained preoperatively.

### Patient Population

We recruited patients (*n* = 96), all with smoking history ≥10 pack-years, scheduled to undergo clinically indicated resections, including for pulmonary nodules, lung volume reduction surgery, or lung transplant, in which case we obtained the explanted tissue. We collected only distal, nonneoplastic lung tissue, lacking postobstructive changes, as judged by a pathologist. Some subjects also contributed peripheral blood 3–6 wk before the surgery. We categorized subjects using the fixed ratio approach recommended by the Global Initiative for Chronic Obstructive Lung Disease (GOLD) (17). Thus, participants (*n* = 37) with a ratio of forced expiratory volume in 1 s to forced vital capacity (FEV_1_/FVC) >0.70, normal spirometry, and no clinical diagnosis of COPD were considered subjects with NAO. Participants (*n* = 59) with FEV_1_/FVC <0.7 were considered to have COPD. Table 1 shows demographics, pulmonary function, and inhaled corticosteroid (ICS) usage.

**Table 1.** Summary of subject demographics, smoking history, and spirometry

Group	NAO Smoker	COPD	*P* Value
Subjects, *n*	37	59	
Sex ratio, M, F	27, 10	44, 15	NS
Age, yr (SD)	62 (9)	64 (7)	NS
Smoking, pack-years (SD)	44 (25) *n* = 1#	58 (31) *n* = 1#	<0.03
Smoking status* (active, former)	13, 23 *n* = 1#	21, 36 *n* = 2#	NS
FEV_1_, % pred. (SD)	94 (15) *n* = 1#	60 (29) *n* = 1#	<0.0001
DLCO, % pred. (SD)	80 (16) *n* = 14#	63 (26) *n* =19#	0.005
ICS usage (yes, no)	2, 35	29, 28 *n* = 2#	<0.0001
Cancer as the indication for surgery (yes, no)	36, 0 *n* = 1#	41, 17 *n* = 1#	0.0002

Values are means (SD), except for sex, smoking status, and inhaled corticosteroids (ICSs), which are presented as counts. DLCO, diffusing capacity of the lung for carbon monoxide; F, female; FEV_1_, forced expiratory volume in 1 s; M, male; NAO, no airway obstruction; pred., predicted; #, missing data from indicated number of participants; *, former smokers, defined as having quit for at least 6 mo.

### Experimental Design and Sample Isolation

We processed lung samples in either of three ways depending on the specific goal, in some cases dividing the sample for several types of processing. Not all experiments were performed on each specimen. First, for flow cytometry, tissues were dispersed using a Waring blender without enzyme treatments, which we have previously shown produces single-cell suspensions of high viability (14, 18,19). Cells were filtered sequentially through cell strainers of 70 µm and then 30 µm pore size to remove debris and then resuspended in staining buffer (2% fetal bovine serum in phosphate-buffered saline). Details of the flow cytometry staining procedure are described below in “*Flow Cytometry of cDC Recovered from Whole Lung Tissue*”.

Second, for cytotoxicity assays, single-cell suspensions prepared as described earlier were subjected to a combination of negative and positive selection using MACS technology (Miltenyi Biotec, Auburn, CA) to produce populations of lung NKs (CD56+ microbeads), lung epithelial cells [CD326+ epithelial cell adhesion molecule (EpCAM) microbeads], and lung DCs, as described previously (7). Lung DCs were either isolated using the Pan-DC Enrichment Kit or, to isolate DC subsets, the CD1c [blood dendritic cell antigen (BDCA)-1] Dendritic Cell Isolation Kit or CD141 (BDCA-3) Microbead Kit (Miltenyi Biotec).

Third, for immunohistochemistry, pieces of human lung tissue were rinsed in RPMI and injected with a 50%:50% mixture of PBS and optimal cutting temperature (OCT) compound, then placed in a tissue cassette, covered with OCT, and snap-frozen before storing in a −80°C freezer.

From participants who additionally agreed to a preoperative blood collection, we cryopreserved peripheral blood NKs, so that they could be used in experiments once their surgical specimen was available. From 10 mL of whole blood collected in heparinized tubes, we immediately isolated CD56+ NKs using the same magnetic sorting techniques described earlier. Isolated NKs were resuspended in 500 µL of FBS and then an additional 500 µL (400 µL FBS plus 100 µL DMSO) was added dropwise. Samples were stored at −80°C until use. Blood NKs were recovered from cryopreservation by thawing at 37°C and then adding to 5 mL of prewarmed RPMI. Cells were allowed to rest at 37°C and 5% CO_2_ overnight before being used as a source of unprimed NKs in experiments involving coculture with autologous lung DCs and lung epithelial cells.

### Flow Cytometry of cDC Recovered from Whole Lung Tissue

Cells were incubated with Live/Dead Fixable Near-IR Dead Cell Stain Kit (Thermo Fisher) according to the manufacturer’s instructions. After washing, cells were incubated with Fc Receptor (FcR) blocking reagent (Miltenyi Biotec, Auburn, CA) for 10 min and then stained in a single tube with monoclonal antibodies against the following antigens (clones, fluorochrome, and vendor shown in parentheses): CD45 (2D1, AmCyan, BD Biosciences), CD3 (SK7, APC-Cy7, eBioscience), CD19 (SJ25C1, APC-Cy7, eBioscience), human leukocyte antigen - DR isotype (HLA-DR) (LN3, PE-Cy7, Biolegend), BDCA-1/CD1c (AD5-8E7, PE, Miltenyi Biotec), C-C chemokine receptor 7 (CCR7) (150503, FITC, R&D Systems), CCR2 (48607, Biotin, R&D Systems), and C-X3-C chemokine receptor 1 (CX3CR1) (2A9-1, APC, Biolegend). In a duplicate tube, the same antibodies were used except for BDCA-1, which was replaced with BDCA-3/CD141 (REA674, PE, Miltenyi Biotec). Cells were incubated on a shaker for 25 min at room temperature while protected from light and then were washed with staining buffer. Cells were resuspended in 2% paraformaldehyde in staining buffer and stored at 4°C. Data were acquired using FACS Diva software on a customized LSR II flow cytometer (BD Biosciences, San Jose, CA), using a three-laser configuration: 488 nm blue laser, 405 nm violet laser, and 633 nm red laser. Data were analyzed using FlowJo Software (BD Biosciences). Details of the gating strategy are described in the results section. A minimum of 10,000 CD45+ events were collected per sample.

### Detection of Airway Epithelial Cell Apoptosis

Frozen lung tissue was sectioned into 20-µm slices using a Cryostat and processed for immunohistochemistry, with dilutions of all reagents made using PBS. Lung sections were fixed in acetone for 5 min at room temperature and then blocked with a solution of PBS and 12.5% FcR blocking reagent for 15 min at room temperature. Next, sections were incubated with antibodies against activated Caspase-3/7 (1:500; PE-labeled; Thermo Fischer Scientific) and EpCAM (1:25; FITC-labeled; Miltenyi Biotec) for 1 h at room temperature, followed by two washes in PBS. Finally, slides were affixed with coverslips using ProLong Gold Antifade Mountant with DAPI (Thermo Fisher Scientific). Slides were viewed using an Olympus BX51W1 confocal microscope equipped with five lasers (555 nm, 488 nm, 450 nm, QD655, and Cy5) running Stereo Investigator version 10.2 software (MBF Bioscience). Images were captured using a Hamamatsu digital camera and an MBF Q imaging camera and were superimposed using Micro-Manager Open Source Microscopy Software. An observer, blinded to participant identity, quantified EpCAM+ epithelial cells located in small airways (and not in alveolar tissues) that were positive for activated Caspase-3/7, by obtaining counts from 40 airways per subject (10 airways from each of 4 separate lung sections).

### DC/NK Coculture and Killing Assay

Viable lung DCs and autologous peripheral blood NKs were resuspended in culture media (20). Pan-DCs and blood NKs were pretreated with LPS (1 µg/mL) or media for 6 h before mixing at a 1:1 ratio. In separate experiments, cDC1 and cDC2 were cultured with blood NKs at a 1:1 ratio. For all DC/NK cocultures, cells were incubated at 37°C and 5% CO_2_ in a 96-well plate for 18 h. CD326+ epithelial cells were added at a ratio of five NKs to one epithelial cell for an additional 4 h and then collected for flow cytometry.

In lung NK killing assays, NKs were cultured with lung epithelial cells alone or with Concanamycin A (ConA; Sigma Aldrich, St. Louis, MO) at 10 nM. Epithelial cells were also cultured alone or with ConA in the absence of NKs. After 4 h, cells were collected for flow cytometry to detect apoptosis using Annexin-V and 7-aminoactinomycin (7-AAD). First, a monoclonal antibody against CD45 was added, and cells were incubated in the dark, with shaking, for 25 min. Cells were washed with 2 mL of staining buffer. Cells were then stained using the Annexin-V and 7-AAD Apoptosis Detection Kit (BD Biosciences, San Jose, CA) according to the manufacturer’s instructions. Cells were analyzed immediately by flow cytometry. Data were acquired using FACS Diva software on a customized LSR II flow cytometer (BD Biosciences, San Jose, CA), using a four-laser configuration: 488 nm blue laser, 405 nm violet laser, 561 nm yellow-green laser, and 633 nm red laser (21). Data were analyzed using FlowJo Software (BD Biosciences). The percentage of cytotoxic cells was determined by the following equation: (Target alone) − (Target + Effector)/Target alone.

### Real-Time PCR

RNA isolation kits (Qiagen, Germantown, MD) were used to isolate RNA from purified lung NKs. Each RNA sample was reverse transcribed using SuperScript II RNase H^−^ Reverse Transcriptase (Thermo Fisher Scientific). Analysis of the transcripts was performed by real-time PCR using a LightCycler480 (Roche). Human GAPDH, which acted as the endogenous reference, and primer-probe sets for perforin (Hs00169473_m1), granzyme A (Hs00989184_m1), granzyme B (Hs00188051_m1), FasL (Hs00899442_m1), TNF-related apoptosis inducing ligand (TRAIL; Hs00921974_m1), and TNF-α (Hs00174128_m1) were purchased commercially (Applied Biosystems). Transcript levels are expressed as arbitrary units and were calculated using the comparative threshold cycle method.

### Statistics

Statistical analyses were performed using GraphPad Prism 7.0 (GraphPad Software, Inc., La Jolla, CA). For data that were not normally distributed, we used Mann–Whitney *U* tests or Friedman ANOVA to determine significance of differences between two groups or three or more groups, respectively. For data that were normally distributed, we used one-way ANOVA to determine differences of three or more groups, and correlations were tested using Spearman regression. The statistical test and specific post hoc analyses are indicated in the figure legends. A two-tailed *P* value of <0.05 indicated significance.

## RESULTS

### Lung NKs in COPD Express More Perforin, by Which They Kill Autologous Lung Epithelial Cells

**Figure 1.**
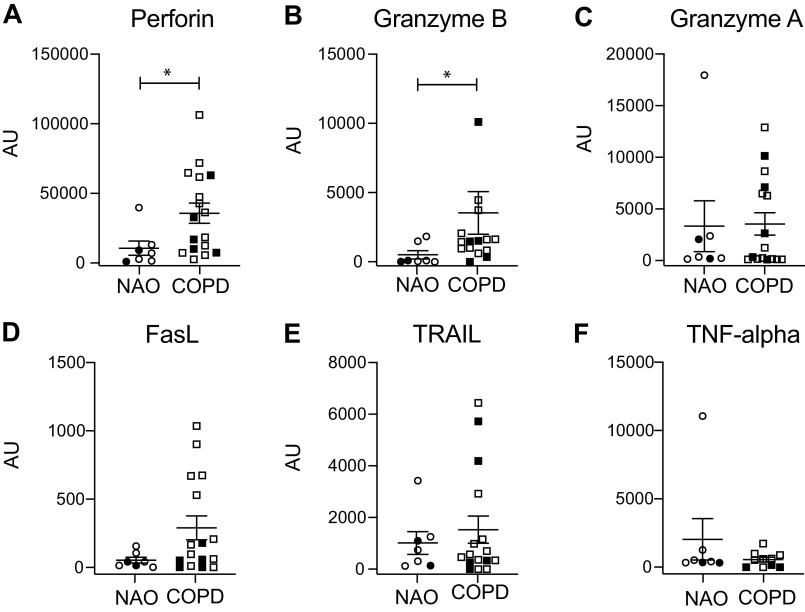
Lung NKs from subjects with COPD have increased transcripts for perforin and granzyme B. Lung tissue was dispersed and CD56+ cells were isolated via magnetic selection. RNA was isolated and transcripts for perforin (*A*), granzyme B (*B*), granzyme A (*C*), FasL (*D*), TRAIL (*E*), and TNF-α (*F*) were measured by real-time PCR by the comparative threshold cycle method, relative to GAPDH. Data are arbitrary units (AU) on the vertical axis, shown as means ± standard error of the mean (SEM); *n* = 7 smokers with no airway obstruction (NAO) for *A*–*F*. *n* = 16 smokers with COPD for *A–E*, and *n* = 10 smokers with COPD for *F*. The Mann–Whitney *U* test was used to determine significance. *P < 0.05. Closed symbols, current smokers; open symbols, former smokers. COPD, chronic obstructive pulmonary disease; FasL, Fas Ligand; TRAIL, TNF-related apoptosis-inducing ligand.

We have previously shown that lung NKs are cytotoxic toward their own autologous epithelial cells and that this cytotoxicity is increased when lung NKs come from subjects with COPD, as compared with smokers without lung obstruction (subjects with NAO) (7). It is well known that NKs can kill targets either through the directed release of lytic granules or by inducing death receptor-mediated apoptosis, but the mechanism used in COPD is unknown. We isolated lung NKs from subjects with NAO and those with COPD and used real-time PCR to measure transcripts for known cytotoxic molecules expressed by NKs. Results showed increased expression of perforin and granzyme B transcripts by lung NKs from participants with COPD (Fig. 1). Transcripts for granzyme A, Fas Ligand, TNF-related apoptosis-inducing ligand (TRAIL), and TNF-α did not differ significantly between subject groups.

**Figure 2.**
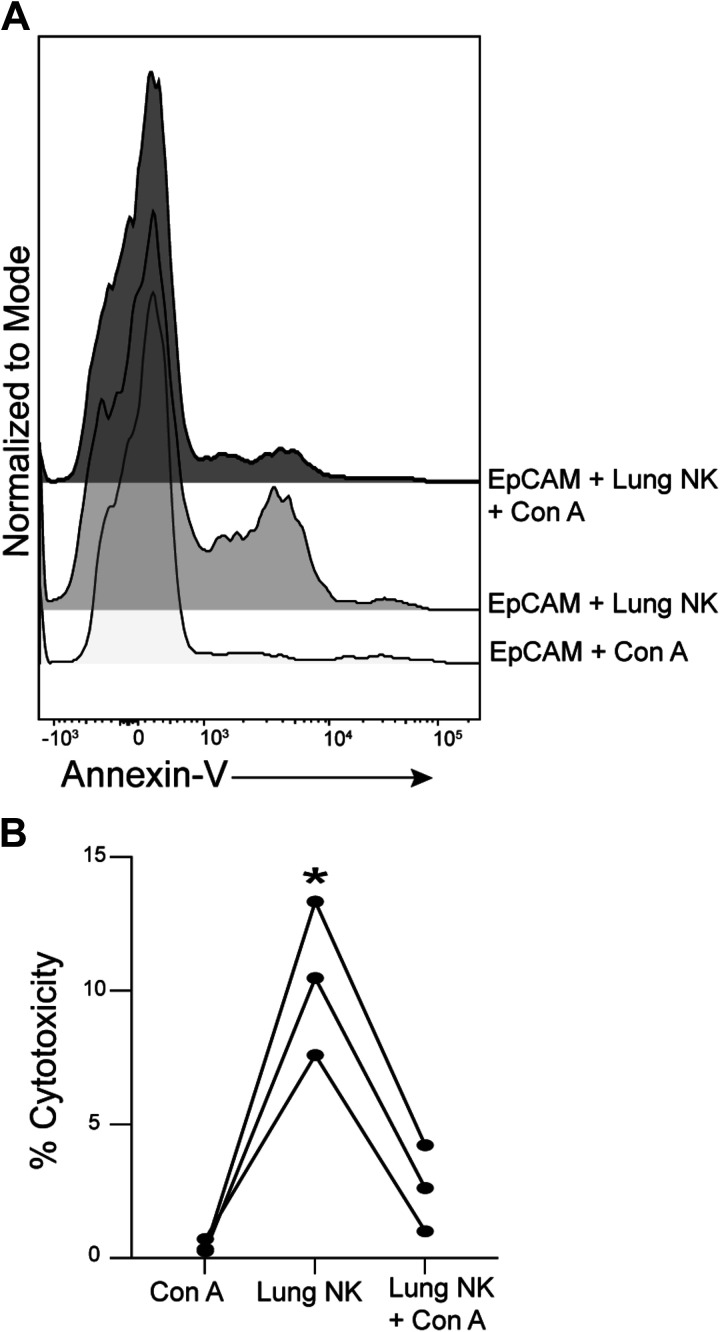
Inhibiting perforin prevents NK-mediated cytotoxicity of lung epithelial cells. Human lung tissue from subjects with COPD (*n* = 3) was dispersed in independent experiments and CD56+ NK cells and EpCAM+ epithelial cells were isolated. NK cells were cocultured with autologous lung epithelial cells in the absence or presence of the perforin inhibitor, Concanamycin A (Con A). As a control, epithelial cells were cultured with Con A without NK cells present. After 4 h, cells were collected and stained with CD45, Annexin-V, and 7-AAD for flow cytometry. Epithelial cells were identified as CD45^−^ cells with a high side scatter. *A*: representative histograms show Annexin-V positivity of epithelial cells. *B*: combined data from all subjects. *P < 0.05 compared with all other conditions as determined by the repeated-measures one-way ANOVA with Tukey’s multiple comparison test. COPD, chronic obstructive pulmonary disease; NK, natural killer cells; 7-AAD, 7-aminoactinomycin.

Because perforin and granzymes synergize to mediate target cell apoptosis, these data suggest that lung NKs use that pathway to kill autologous lung epithelial cells. To support that possibility, we cocultured lung NKs from subjects with COPD with autologous lung epithelial cells in the presence or absence of the perforin inhibitor Concanamycin A (ConA). NK cytotoxicity was significantly decreased by ConA (Fig. 2), which had no effect on epithelial cells alone, suggesting that the release of perforin and granzyme is likely responsible for NK-mediated killing of lung epithelial cells in COPD.

### In Vitro Cytolysis by Lung NKs Correlates with Epithelial Cell Apoptosis in Situ

**Figure 3.**
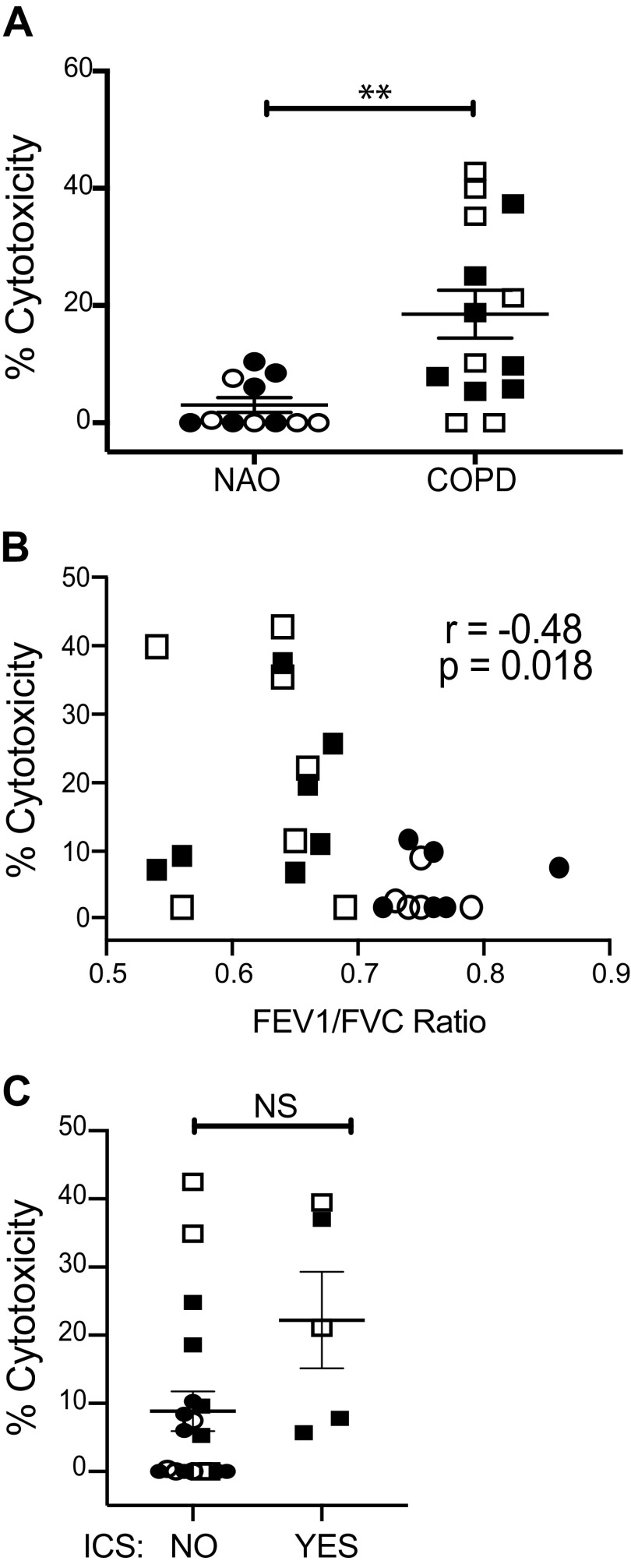
Relative to smokers without airflow obstruction, lung NK cells from subjects with COPD are more cytotoxic toward autologous lung epithelial cells. Lung NKs from subjects with NAO and those with COPD were cocultured either alone or with autologous lung epithelial cells for 4 h. *A*: cytotoxicity was measured via Annexin-V and 7-AAD staining; *n* = 11 NAO and *n* = 14 COPD. *B*: percent cytotoxicity vs. FEV_1_/FVC ratio for individual subjects. *C*: percent cytotoxicity separated by ICS use. The Mann–Whitney *U* test was used to determine significance for *A* and *C*. Spearman regression was used to test correlation (*B*). Circles, subjects with NAO; squares, subjects with COPD; closed symbols, current smokers; open symbols, former smokers. In *A* and *C*, lines represent the means ± SEM. **P < 0.01. COPD, chronic obstructive pulmonary disease; FEV_1_/FVC, forced expiratory volume in one second/forced vital capacity; ICS, inhaled corticosteroids; NAO, no airway obstruction; NS, not significant.

We measured the cytotoxicity of NK cells toward autologous lung epithelial cells. In agreement with our previous findings (7), but in an independent cohort, NKs showed significantly higher cytotoxicity (*P* < 0.006) toward lung epithelial cells in subjects with COPD compared with subjects with NAO (Fig. 3*A*). Increased NK cytotoxicity correlated with decreasing FEV_1_/FVC (*r* = −0.48; *P* = 0.018) (Fig. 3*B*), but not FEV_1_% predicted, also consistent with previous results (7). All the participants used in Fig. 3, except one in the COPD group, underwent clinically indicated resections due to cancer. Because subjects with COPD were more likely to be taking inhaled corticosteroids (ICSs), which might be immunomodulatory, we examined the effect of such therapy on lung NK cytotoxicity. There was nonsignificant difference that was greater in subjects taking ICSs (22.2 ± 15.8% vs. 8.8 ± 12.7%) (Fig. 3*C*), perhaps reflecting confounding by indication. Importantly, all subjects with COPD had either mild (GOLD I) or moderate (GOLD II) spirometric severity, implying that elevated NK cytotoxicity is not limited to severe or end-stage disease.

**Figure 4.**
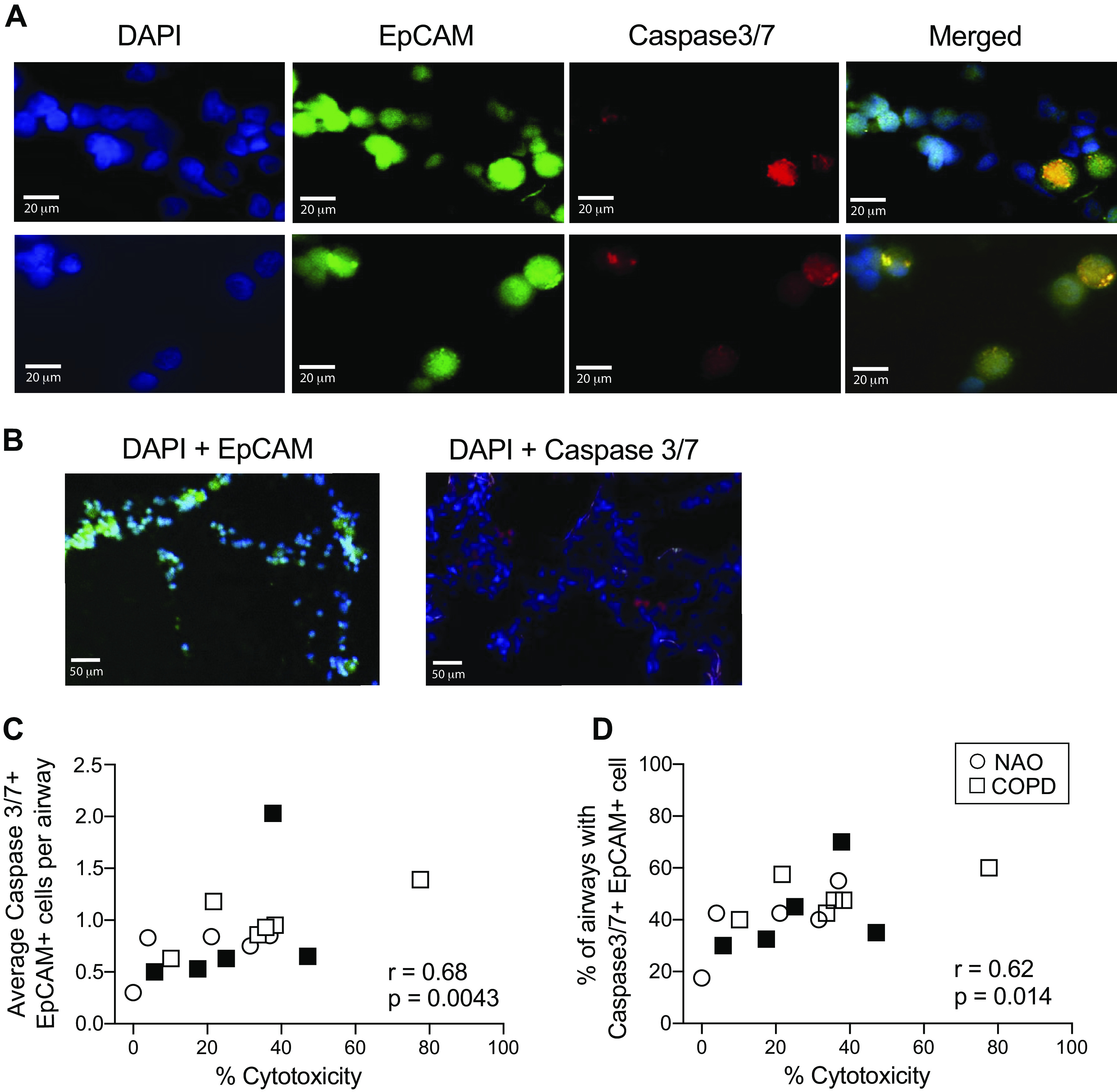
The magnitude of epithelial cell apoptosis in vivo correlates with observed NK cytotoxicity in vitro. Human lung tissue was divided in half and was either frozen to detect epithelial apoptosis in situ or processed to obtain viable cells for the flow cytometric analysis of in vitro NK killing. Frozen tissues were sectioned and stained with DAPI and antibodies against EpCAM and activated Caspase-3/7. The number of cells per airways that were double-positive for both EpCAM and activated Caspase-3/7 was counted. *A*: representative staining from two subjects. Blue, DAPI; green, EpCAM; red, activated Caspase-3/7; merged cells that are positive for DAPI, EpCAM, and Caspase-3/7 will appear yellow; magnification = ×40. *B*: staining for DAPI + EpCAM (control for Caspase-3/7) or DAPI + Caspase-3/7 (control for EpCAM); magnification = ×10. *C*: the average number of Caspase-3/7+ EpCAM+ cells per airway correlated to the percent cytotoxicity determined in vitro. *D*: the percentage of airways that had one or more Caspase-3/7+ EPCAM+ cells also positively associated with the corresponding percent cytotoxicity. Closed symbols, current smokers; open symbols, former smokers. Circles, participants with no airway obstruction (*n* =5); squares, participants with COPD (*n* = 11). The Spearman nonparametric correlation was used to determine significance. COPD, chronic obstructive pulmonary disease; NAO, no airway obstruction.

We next wanted to determine the biological relevance of our in vitro NK cytotoxicity assay to disease processes occurring in vivo. For some subjects, we obtained sufficient lung tissue to perform both the in vitro cytotoxicity assay and to assess epithelial cell apoptosis in intact frozen lung tissue. To determine whether we could detect evidence of epithelial cell apoptosis in vivo, we stained frozen lung tissue for activated Caspase-3/7, a reliable marker of apoptosis that is also known to be catalyzed by granzyme B (22), and analyzed them by confocal microscopy. Representative staining from two subjects is shown (Fig. 4*A*; magnification = ×40). Spearman analysis showed a strong correlation between in vitro cytotoxicity and in situ apoptosis, whether expressed as the average number of Caspase-3/7+ EpCAM+ cells per airway (*S_r_* = 0.68, *P* = 0.0043) (Fig. 4*C*) or as the percentage of airways containing one or more apoptotic epithelial cells (*S_r_* = 0.62, *P* = 0.014) (Fig. 4*D*). These data provide evidence that our NK cytotoxicity assay reflects the epithelial cell apoptosis occurring in vivo in COPD.

### Lung DCs from Subjects with COPD Have Increased Expression of CCR7 but Not CCR2 or CX3CR1

**Figure 5.**
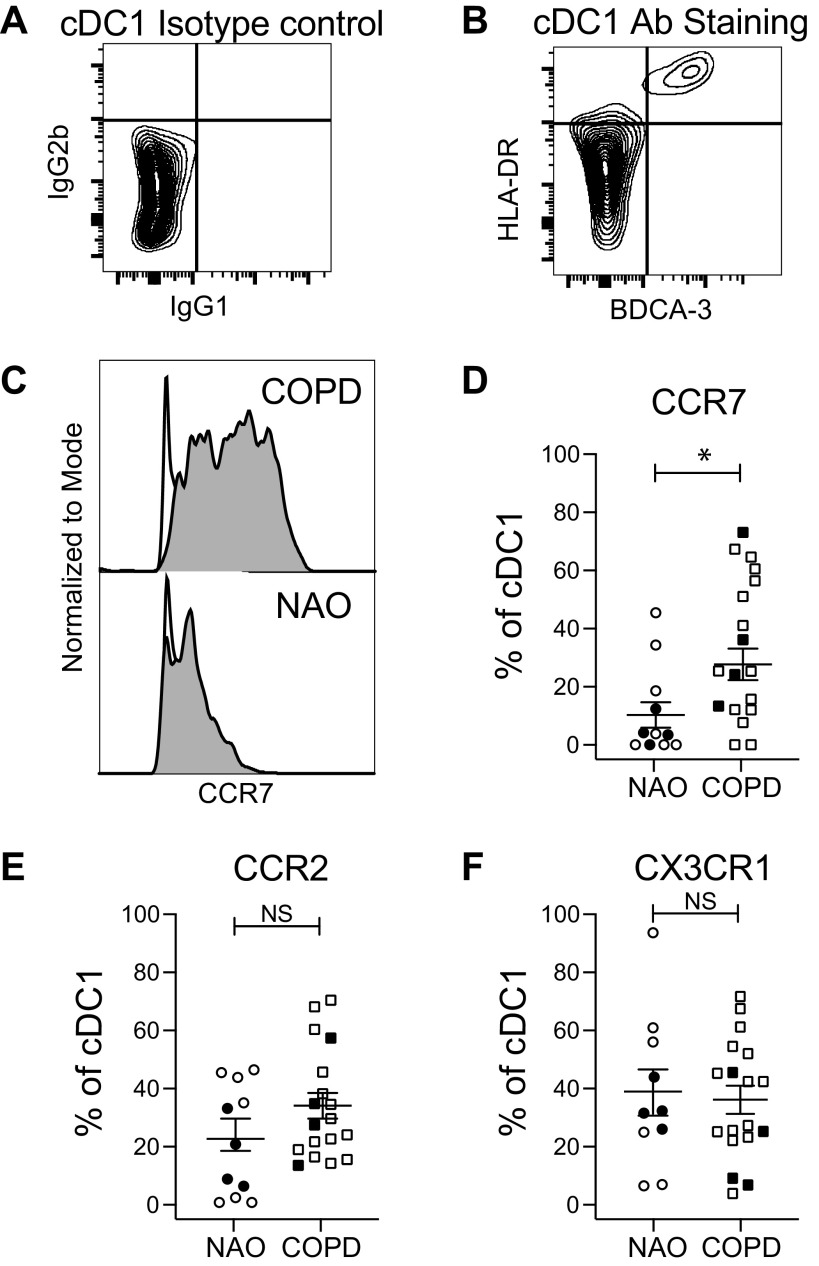
Identification of lung cDC1 and chemokine receptor expression. Single-cell suspensions of dispersed human lung tissue were stained with fixable live-dead stain and antibodies toward CD45, CD3, CD19, BDCA-3, HLA-DR, CCR7, CCR2, and CX3CR1. After gating on cells that were viable, CD45+, CD3−, and CD19−, cDC1 cells were identified by comparing an isotype control (*A*) with antibody staining (*B*) for HLA-DR and BDCA-3. *C*: representative staining showing the expression of CCR7 on cDC1 cells from subjects with NAO and those with COPD; white histogram, isotype control; gray histogram, CCR7 staining. *D*–*F*: compiled data from all subjects for the percentage of cDC1 expressing CCR7 (*D*), CCR2 (*E*), and CX3CR1 (*F*). Symbols indicate individual participants; circles, participants with NAO (*n* = 11); squares, participants with COPD (*n* = 18). Lines represent the means ± SEM. The Mann–Whitney *U* test was used to determine significance; *P < 0.05. Closed symbols, current smokers; open symbols, former smokers. cDC1, conventional dendritic cell, type 1; COPD, chronic obstructive pulmonary disease; NAO, no airway obstruction; NS, not significant.

We previously showed that lung DCs are required to prime NKs to become cytotoxic and that coculture with lung DCs from subjects with COPD induces greater cytotoxicity in autologous blood NK cells, relative to the same design in subjects with NAO (7). To gain an understanding of this difference, we first extended our previous finding that lung DCs in COPD have increased expression of markers of maturation and activation, including CD40, CD80, and CD86 (14), by examining CCR2, CCR7, and CX3CR1, which are chemokine receptors involved in DC maturation and trafficking (23–25). Lung cDC1 were identified by gating on viable, CD45+CD3−CD19− cells, selecting cells double-positive for HLA-DR and BDCA-3 (CD141) (Fig. 5, *A* and *B*) and then analyzing expression of CCR7 (Fig. 5, *C* and *D*), CCR2 (Fig. 5*E*), and CX3CR1 (Fig. 5*F*).

**Figure 6.**
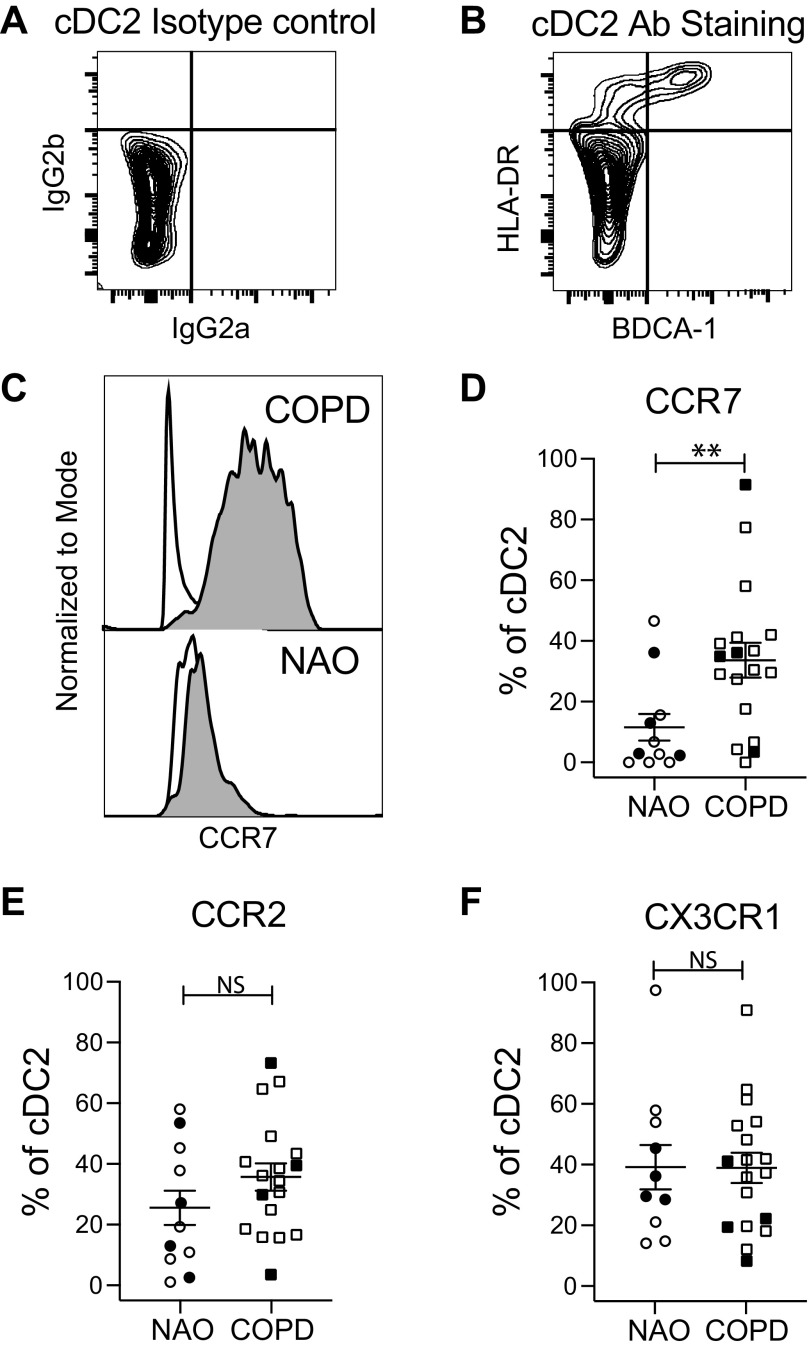
Identification of lung cDC2 and chemokine receptor expression. Single-cell suspensions of dispersed human lung tissue were stained with fixable live-dead stain and antibodies toward CD45, CD3, CD19, BDCA-1, HLA-DR, CCR7, CCR2, and CX3CR1. After gating on cells that were viable, CD45+, CD3−, and CD19−, cDC2 cells were identified by comparing an isotype control (*A*) with antibody staining (*B*) for HLA-DR and BDCA-1. *C*: representative staining showing the expression of CCR7 on cDC2 cells from subjects with NAO and those with COPD; white histogram, isotype control; gray histogram, CCR7 staining. *D–F*: compiled data from all subjects for the percentage of cDC2 expressing CCR7 (*D*), CCR2 (*E*), and CX3CR1 (*F*). Symbols indicate individual participants; circles, participants with NAO (*n* = 11) and squares, participants with COPD (*n* = 18). Lines represent the means ± SEM. The Mann–Whitney *U* test was used to determine significance; **P < 0.01. Closed symbols, current smokers; open symbols, former smokers. cDC2, conventional dendritic cell, type 2; COPD, chronic obstructive pulmonary disease; NAO, no airway obstruction; NS, not significant.

A similar approach was taken for cDC2, selecting cells that were double-positive for HLA-DR and BDCA-1 (CD1c) (Fig. 6, *A* and *B*) and then analyzing chemokine receptor expression (Fig. 6, *C*–*F*). The percentage of both cDC1 and cDC2 expressing CCR7 was increased in subjects with COPD compared with subjects with NAO. Expression of CCR2 or CX3CR1 did not differ between subject groups for either cDC subset. Because CCR7 is essential for DC emigration to lymph nodes, these data extend the evidence of lung DC maturity in COPD.

### Maturation of Lung DCs by LPS Stimulation Drives Increased Priming of Autologous NKs

**Figure 7.**
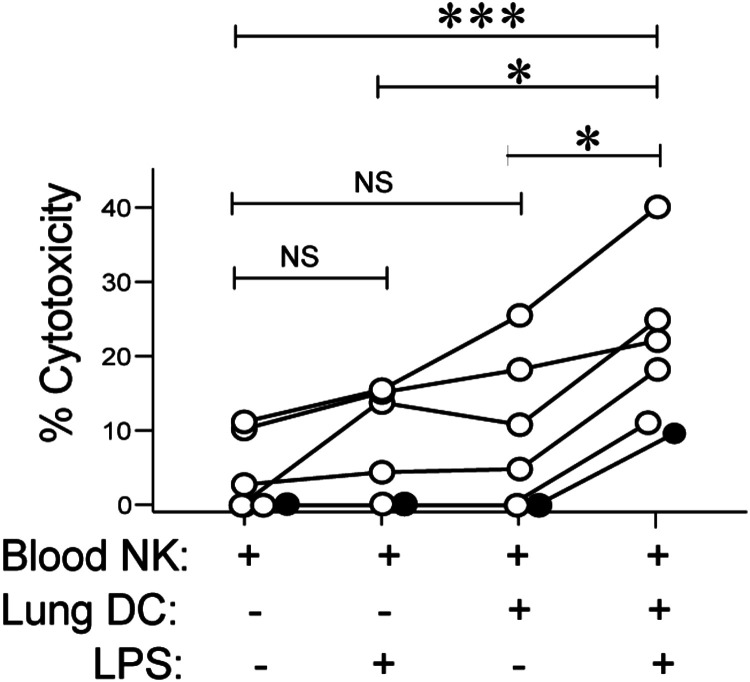
Maturation by LPS pretreatment increases the ability of lung DCs from smokers without COPD to prime blood NK cells for cytotoxicity. Human lung tissue and peripheral blood was collected from subjects with NAO (*n* = 6). Magnetic beads were used to isolate CD56+ cells from blood and pan-DCs and EpCAM+ epithelial cells from lung tissue. Lung DCs were pretreated without or with LPS for 6 h, then cocultured with blood NK cells for 18 h, and finally EpCAM+ lung epithelial cells were added for an additional 4 h. Cells were collected and stained with CD45, Annexin-V, and 7-AAD for flow cytometry. Epithelial cells were identified as CD45− with a high side scatter. The percent cytotoxicity is the decrease in viable cells compared with epithelial cells cultured alone to measure spontaneous apoptosis. The Friedman ANOVA with two-stage linear step-up procedure of Benjamin, Krieger, and Yekutieli was used to determine significance between groups. *P < 0.05, ***P < 0.001. Closed symbols, current smokers; open symbols, former smokers. COPD, chronic obstructive pulmonary disease; DC, dendritic cell; NAO, no airway obstruction; NK, natural killer cell; NS, not significant; 7-AAD, 7-aminoactinomycin.

Although the increased maturity of lung cDC in COPD might explain their superior NK priming (26), it is also possible that smokers whose cDC intrinsically lack that capacity are protected from COPD development. Since we have previously shown that blood NKs from both subjects with NAO and those with COPD show a similar lack of cytotoxicity toward autologous lung epithelial cells compared with their lung NK counterparts (2), coculturing blood NKs with autologous lung DCs allows us to test whether priming ability can be induced. Therefore, we pretreated lung DCs from subjects with NAO with LPS or medium alone and then cocultured them with autologous blood NKs, which we assayed for cytotoxicity (Fig. 7). Coculture with untreated lung DCs led to a nonsignificant increase in the cytotoxicity of autologous blood NKs, compared with blood NKs alone, in agreement with our published data (7). Results were similar when blood NKs received LPS stimulation in the absence of DCs, in agreement with murine data showing that priming is mediated via DCs and not by direct activation of NK cells (11, 27, 28). However, LPS pretreatment of DCs was associated with a greater than fourfold increase in NK cytotoxicity, significantly greater than other conditions (Fig. 7). These results have several implications. They imply that the low level of killing of autologous lung epithelial cells by lung NK cells does not reflect intrinsic resistance of the targets or an inability by the NKs to kill if primed. They also suggest that the paucity of killing in subjects with NAO results from immaturity of their lung DCs.

### Human Lung cDC1 from Subjects with COPD Exclusively Prime Autologous Blood NKs

**Figure 8.**
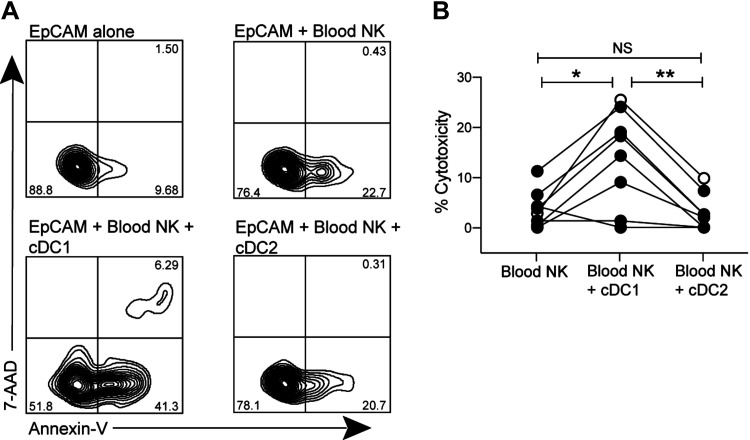
cDC1, but not cDC2, mediate priming of NK cells in COPD. Blood NKs from subjects with COPD were cocultured either alone or with autologous lung cDC1 or cDC2 for 18 h. Autologous lung epithelial cells were added for an additional 4 h, then cytotoxicity was measured via Annexin and 7-AAD staining. *A*: representative Annexin-V (horizontal axis) and 7-AAD (vertical axis) staining of gated epithelial cells cultured with nothing (*top left*), blood NKs (*top right*), blood NKs + cDC1 (*bottom left*), or blood NKs + cDC2 (*bottom right*). *B*: combined data of subjects with COPD (*n* = 8), repeated-measures one-way ANOVA with Tukey’s multiple comparison test. *P < 0.05; **P < 0.01. Closed symbols, current smokers; open symbols, former smokers. COPD, chronic obstructive pulmonary disease; cDC1, conventional dendritic cell, type 1; cDC2, conventional dendritic cell, type 2; NK, natural killer cell; NS, not significant; 7-AAD, 7-aminoactinomycin.

Finally, we investigated which lung cDC subset was responsible for priming NKs in COPD. Blood NKs from subjects with COPD only were cocultured either alone, with cDC2, or with cDC1 for 18 h before adding lung epithelial cells. Representative staining of Annexin-V and 7-AAD after gating on epithelial cells demonstrates the proportion of cells that remain viable (Annexin-V^neg^ and 7-AAD^neg^) or transition to early apoptotic (Annexin-V^pos^ and 7-AAD^neg^) and late apoptotic (Annexin-V^pos^ and 7-AAD^pos^) stages (Fig. 8*A*). Paired data show that blood NKs cocultured with lung cDC1 developed greater cytotoxicity toward lung epithelial cells compared with both blood NKs alone and blood NKs plus cDC2 (Fig. 8*B*). These results demonstrate a unique role for cDC1 in initiating NK responses in COPD.

## DISCUSSION

To investigate the role of human lung DC in priming lung NKs to kill autologous epithelial cells, we analyzed lung tissue resected from 96 ever-smokers with or without COPD. Results demonstrate that *1*) lung NK cytotoxicity is increased in COPD, relative to smokers without airflow obstruction, and is mediated at least in part by the perforin/granzyme pathway; *2*) the percentage of epithelial cell killing determined by our cytotoxicity assay correlated tightly with in situ apoptosis of airway epithelial cells in the same subject; *3*) in COPD, relative to nonobstructed participants, both lung cDC1 and cDC2 displayed increased surface expression of CCR7 (which mediates DC emigration to lymph nodes) but not of CCR2 or CX3CR1 (which mediate DC recruitment into the lungs); *4*) the modest ability of lung DC from nonobstructed participants to prime blood NKs for cytotoxicity was increased by LPS-induced maturation; and *5*) cDC1, but not cDC2, prime blood NKs to kill airway epithelial cells in COPD. These findings provide novel insights into the pathogenesis of smoking-induced COPD, especially its emphysematous phenotype (3, 4, 29).

Previously, using both human lung tissue and two murine models, we showed that lung NK cytotoxicity toward autologous epithelial cells is increased in COPD and depends on IL-15Rα-transpresentation by lung DCs (7). Using a larger, entirely new cohort, we confirm that NK cytotoxicity toward epithelial cells is increased in COPD and extend that finding by demonstrating a strong correlation between our cytotoxicity assay and the detection of airway epithelial cell apoptosis in situ. Our in vitro assay permits dissection of molecular mechanisms of NK priming, as illustrated by greater priming by lung DCs from nonobstructed smokers following maturation, and of NK cytotoxicity, as in the perforin blockade experiments. Hence, validation that the assay accurately reflects lung pathology sets the stage for future mechanistic studies and preclinical testing of potential therapeutic strategies using human pathological tissues and murine models.

A crucial step in the development of such strategies was achieved by the finding that NKs in COPD were primed only by the lung cDC1 subset. Although lung cDC contribute to COPD pathogenesis (10), the relative roles of cDC1 versus cDC2 have remained uncertain. cDC1 derive uniquely from committed bone marrow precursors under the influence of the transcription factors interferon regulatory factor 8 (IRF8) and basic leucine zipper activating transcription factor (ATF)-like transcription factor-3 (BATF-3), whereas cDC2 are heterogeneous, both ontogenically, as they derive in part from monocytes, and functionally (30, 31). Hence, it might be more straightforward to develop selective therapeutic interventions to prevent destructive priming of NKs by cDC1. However, any such approach would need to be designed thoughtfully and cautiously. cDC1 are necessary to elicit Th1 and cytotoxic T-cell responses for antiviral defenses (32). They also maintain self-tolerance at mucosal surfaces by cross-presenting antigens derived from apoptotic cells to generate peripheral regulatory T cells (T_reg_) (33).

Consistent with their strong expression of CD103, many cDC1 reside in part in airway mucosa, where their dendritic processes contact epithelial cells that influence them by secreting cytokines and other proteins (34). DC maturation is promoted by airway epithelial activation of NF-κB (35), a consequence of smoking-induced loss of small airway mucosal defenses (36–38). Although the maturation of differentiated human DCs was inhibited by nicotine (39), recognition of bacterial molecules via Toll-like receptors (TLRs) reversed that inhibition (40). Breaching of the airway epithelial barrier initiates small airways disease (41), the earliest lesion in COPD (42, 43). Thus, recognition by immature cDC1 of bacterial invasion of the epithelium is a plausible trigger that could ignite multiple destructive processes that lead to COPD pathology. Besides pathogen-associated molecular patterns (PAMPs), cDC1 also mature in response to damage-associated molecular patterns (DAMPs), including sterile injury sensed via TLR3 (44). Epithelial cells exposed to cigarette smoke extract released double-stranded DNA, high-mobility group box 1 (HMGB1), and heat shock proteins (HSPs) (45). HMGB1 also induces DC maturation and activation and increase in the bronchoalveolar lavage fluid of patients with COPD (46). Interestingly, in response to DC-secreted IL-18, NKs themselves release HMGB1, which permits immature DCs to escape NK-mediated lysis (47). Other DAMPS, including β-defensins and HSPs, are also increased in COPD and have a known role in DC recruitment and/or activation (20). Because airway cDC1 are relatively short-lived cells that must be replenished continuously from the bone marrow, defining the interactions that drive activation represents a crucial knowledge gap.

Another important unsettled question is where within the lungs cDC1 and NKs colocalize. We suspect one site is in the airway mucosa, mediated by NK expression of CCR4, which recognizes multiple cDC1-derived chemokines. Supporting this possibility, we previously demonstrated CCR4-mediated localization and activation of NKs within the lungs in a murine smoke-exposure model (48). NK priming by cDC1 might also occur in lung lymphoid follicles (LLFs), which prominently develop within lung parenchyma in more severe stages of COPD (26). However, we now show substantial lung NK cytotoxicity even in subjects with COPD with GOLD stages 1 and 2, suggesting that DC priming can occur in those predicted to have few to no LLFs. A third possibility, in regional lymph nodes, seems less likely given the lack of their hypertrophy in COPD relative to the expansion of LLFs. Our current finding of increased CCR7 expression by both cDC subsets in COPD, relative to nonobstructed smokers, is relevant. Recruitment of DC into both LLFs and lymph nodes depends on CCR7. In a murine model, the inability of CCR7-deficient DCs to leave the lung parenchyma via lymphatic vessels was associated with spontaneous LLF formation (49). That same study found that lung DCs of aged mice had reduced CCR7 expression and impaired emigration (49). However, we detected no correlation between participant age and CCR7 expression (data not shown), nor did ages differ significantly between our groups. Hence, our results imply that development of LLFs, recently shown to depend on cDC2 (50), is unlikely to result from impaired cDC emigration from the lungs. An acknowledged limitation is our inability to study regional lymph nodes, which are needed in entirety by the clinical pathologists for diagnostic analysis.

Our data supporting a role for perforin and granzyme in NK-mediated killing implicate that pathway as a potential target to prevent emphysema. Lytic granule release is tightly regulated and directed, preventing diffusion to neighboring cells (22). A minority of NKs are responsible for most killing events (51), and individual NKs can kill multiple consecutive target cells, as they release only ∼10% of their cytotoxic granules into a single target (52). However, much of the previous work on which those data are based was conducted using tumor cells as targets, so whether these properties pertain in COPD and autologous target cells remains undefined. Surprisingly, individual NKs that exhaust their lytic granules supplies can switch from perforin/granzyme-mediated killing to death receptor-mediated killing (53). It is unlikely that the NKs in our cytotoxicity assay would have had an opportunity to switch killing modalities given the short 4-h time frame, but we cannot exclude that possibility in vivo.

Our study has several other limitations, largely stemming from our exclusive use of human lung tissue resected for clinical indications. We evaluated only cDC1 and cDC2, and so cannot exclude NK priming by plasmacytoid DCs, which, however, has been shown not to occur in mice (11). We did not show priming of NKs by bulk cDC2, but recent single-cell RNA-seq studies have shown that cDC2 comprise two different subsets (cDC2A and cDC2B) that have opposing roles in tolerance (cDC2A) and inflammation (cDC2B) (30, 31). Hence, it remains possible that further dissection could disclose a role for a subset of cDC2 in NK priming. Finally, intriguing questions about DC-NK interactions, raised by our experiments involving LPS-stimulated blood NKs from subjects with NAO, merit future investigation. Our previous work shows that blood NKs from subjects with COPD are not universally maximally primed, as their cytotoxicity can be increased by coincubation with autologous lung DCs (7); however, whether the priming activity by COPD lung DCs can also be further boosted is unproven but likely. Determining the range of stimuli that can activate DCs to prime NK cytotoxicity would be interesting but is beyond the scope of this study.

In conclusion, we show a central role for lung NKs in emphysema pathogenesis by correlating their in vitro cytotoxicity with Caspase-3/7 activation measured in situ. We also demonstrate a role for mature lung DCs in this process and identify cDC1 as the necessary DC subset driving NK priming in COPD.

## GRANTS

This work was supported in part by Merit Review Awards I01 CX001553 (to C.M.F.) and I01 CX000911 (to J.L.C.) from the Department of Veterans Affairs. These investigations were also supported in part by the Tissue Procurement Core of the University of Michigan Comprehensive Cancer Center, Grant No. P30 CA46952.

## DISCLOSURES

J.L.C. reports grants from National Heart, Lung, and Blood Institute, the Department of Veterans Affairs, and the Department of Defense and personal fees from Astra Zeneca, CSL Behring, and Novartis, outside the scope of this study. C.M.F. reports grants from National Heart, Lung, and Blood Institute and the Department of Veterans Affairs. None of the other authors has any conflicts of interest, financial or otherwise, to disclose. 

## AUTHOR CONTRIBUTIONS

J.L.C. and C.M.F. conceived and designed research; A.M.P., J.X.R., D.T.M., M.S.M., V.R.S., A.T., L.M., and C.M.F. performed experiments; A.M.P., J.X.R., M.S.M., M.S.T., V.R.S., A.T., and C.M.F. analyzed data; M.S.T., V.R.S., J.L.C., and C.M.F. interpreted results of experiments; C.M.F. prepared figures; C.M.F. drafted manuscript; A.M.P., J.X.R., D.T.M., M.S.M., M.S.T., V.R.S., A.T., L.M., J.L.C., and C.M.F. edited and revised manuscript; A.M.P., J.X.R., D.T.M., M.S.M., M.S.T., V.R.S., A.T., L.M., J.L.C., and C.M.F. approved final version of manuscript. 
